# Serum Human Chorionic Gonadotropin (β- hCG) Clearance Curves in Women with Successfully Expectantly Managed Tubal Ectopic Pregnancies: A Retrospective Cohort Study

**DOI:** 10.1371/journal.pone.0130598

**Published:** 2015-07-02

**Authors:** Samir Helmy, Dimitrios Mavrelos, Elinor Sawyer, Jara Ben-Nagi, Marianne Koch, Andrea Day, Davor Jurkovic

**Affiliations:** 1 Department of Obstetrics and Gynecology, Medical University of Vienna, Vienna, Austria; 2 Early Pregnancy and Gynaecology Assessment Unit, King’s College Hospital, London, United Kingdom; 3 Karl Landsteiner Society, Vienna, Austria; 4 Gynaecology Diagnostic and Outpatient Treatment Unit, University College Hospitals London (UCL), London, United Kingdom; Hokkaido University, JAPAN

## Abstract

**Objective:**

To establish clearance curves for serum β -hCG in women with successfully expectantly managed tubal ectopic pregnancies.

**Design:**

Retrospective cohort study. Non- viable tubal ectopic pregnancy was diagnosed on transvaginal ultrasound. If initial serum β hCG was less than 5000 IU/L and patients were asymptomatic, expectant management was offered. Patients underwent serial β hCG measurements until serum β hCG was less than 20 IU/l, or the urine pregnancy test was negative.

**Setting:**

Early Pregnancy and Gynaecology Assessment Unit, Kings College Hospital, London (December 1998 to July 2006).

**Patients:**

We included 161 women with diagnosed non-viable tubal ectopic pregnancy who underwent successful expectant management.

**Main outcome measure:**

Serum β hCG level.

**Results:**

Mean initial serum β- hCG was 488 IU/L (41 - 4883) and median serum β hCG clearance time was 19 days (5 - 82). The average half-life of β hCG clearance was 82.5 hours (±SD 50.2) in patients with steadily declining serum β- hCG levels compared to 106.7 hours (±SD 72.0) in patients with primarily plateauing β-hCG levels in the declining phase. However, these differences were not significant (p>0.05).

**Conclusion:**

We identified a median follow-up of 19 days until serum β hCG clearance in women with tubal ectopic pregnancy and successful expectant management. Although non- significant, women with initially plateauing serum β hCG showed a longer follow-up time until clearance compared to women with steadily declining β hCG levels. This information may serve as a guideline enabling clinicians to predict the length of follow-up for women with tubal ectopic pregnancy and expectant management.

## Introduction

The first case of planned successful expectant management of ectopic pregnancy (EP) was described by Lund in 1955 [1]. Since then several case series have been published, demonstrating that expectant management is a safe and effective management option for women with small non-viable tubal EPs [2–9]. The chance of success of expectant management of tubal EP depends on the selection criteria [2]. These usually include women with mild clinical symptoms who present with relatively low initial serum βhCG levels. The increasing sensitivity of modern transvaginal ultrasound equipment has enabled the detection of small tubal EPs. Many of these EPs represent tubal miscarriages, which are destined to resolve spontaneously. As a result in some recent studies up to 40% of all tubal EPs were successfully managed without any medical or surgical intervention [2].

The main concern with expectant management of tubal EP is a significant failure rate, which can be as high as 30% [2]. Another important consideration is the length of time that is necessary for serum β hCG clearance. Serum β hCG clearance occurs when no functional trophoblast remains and signifies pregnancy resolution. Information about time required for pregnancy resolution is essential to women to make an informed choice between expectant, surgical and medical treatment of tubal ectopic pregnancy. Although there are data in the literature about the dynamics of serum β hCG in women following successful medical treatment with methotrexate, there is no such data in regards to expectant management [10–12]. The aim of this study is to assess the changes in serum β hCG levels in a large group of women who had successful expectant management of tubal EP without embryonic heartbeat on ultrasound scan. This information can then be used to estimate the length of time necessary for the pregnancy to resolve and help to improve planning of follow up visits.

## Material and Methods

IRB approval was waived by the Ethics Committee (King’s College Hospital, London, UK). This was a retrospective study, where data were anonymized and de-identified prior to analysis. The study was conducted in the Early Pregnancy and Gynaecology Assessment Unit, Kings College Hospital, London. This is a tertiary referral unit, which primarily serves a racially diverse, low socio-economic status, urban population.

All women either self-referred or were referred for assessment by their general practitioners or hospital consultants because of suspected early pregnancy complications. All women, who had a positive urinary pregnancy test, underwent a transvaginal ultrasound examination to assess the location and viability of pregnancy. All examinations were performed by gynaecologists, who were trained in the diagnosis and management of early pregnancy complications.

**Fig 1:**
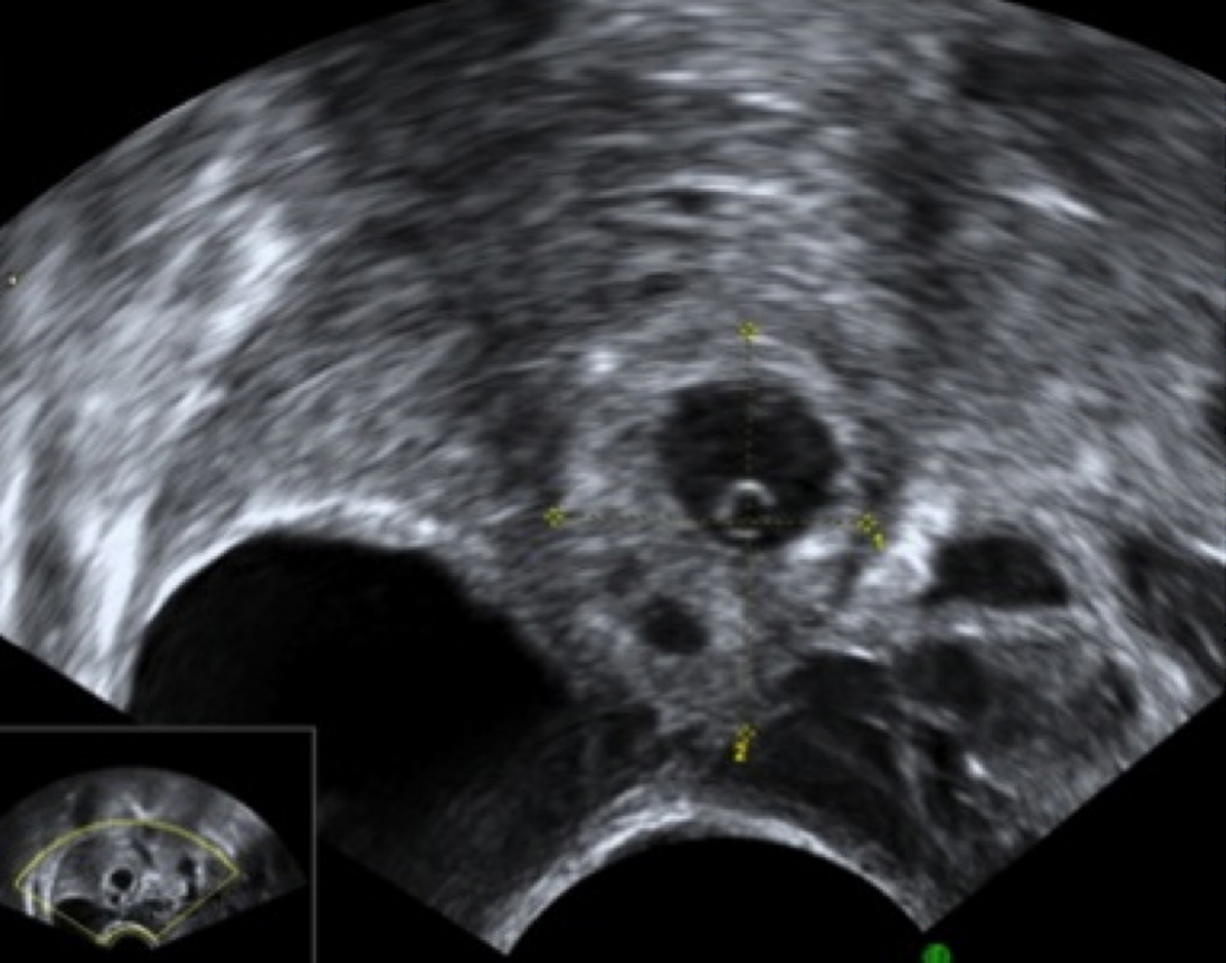
Ultrasound image of a right tubal ectopic pregnancy at 6^+2^ weeks gestation. A gestation sac is seen adjacent to the right ovary.

**Fig 2:**
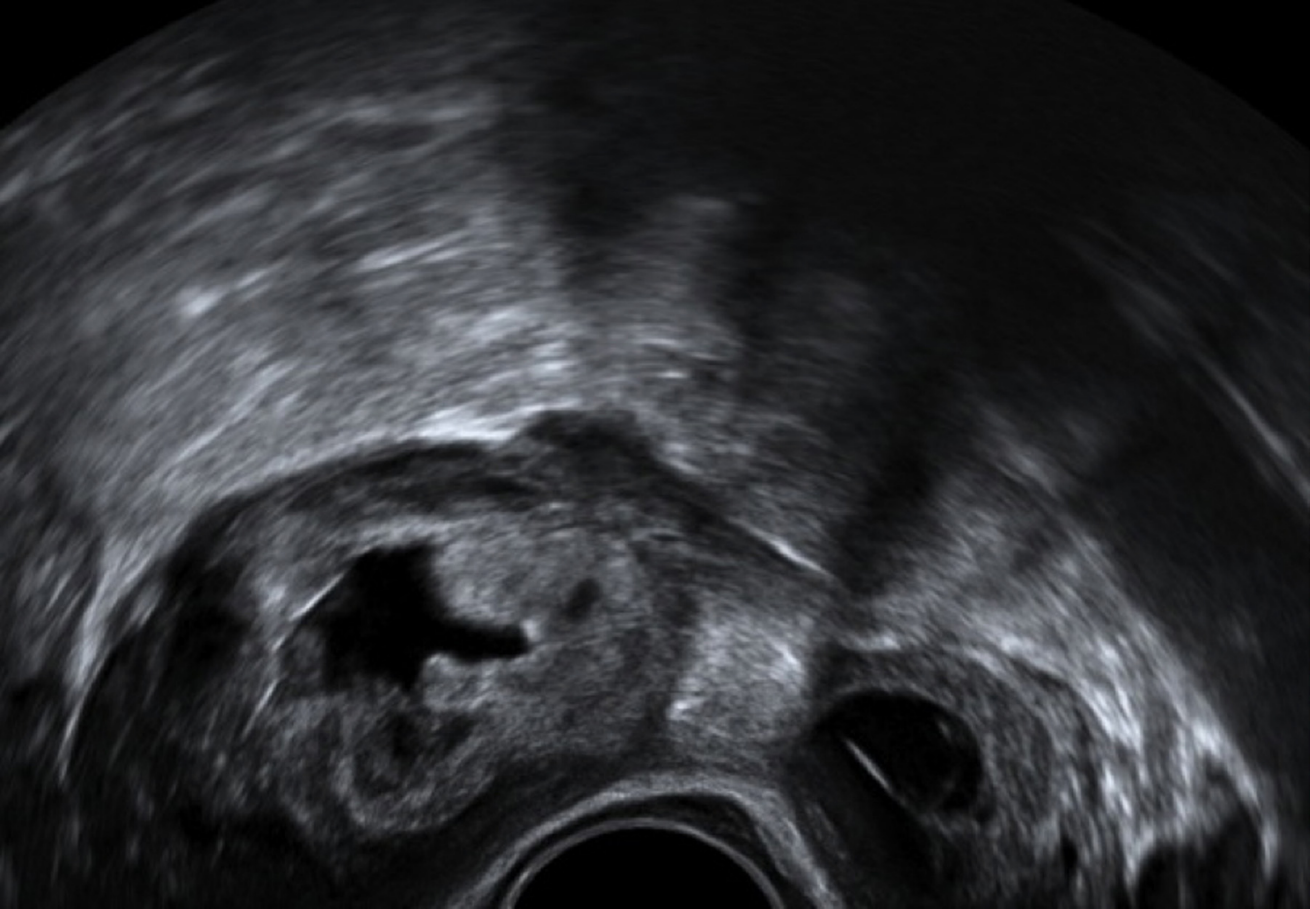
Ultrasound image of a left tubal ectopic at 6+1 weeks gestation. A hyperechoic mass is seen adjacent to the left ovary.

The ultrasound criteria for the diagnosis of ectopic pregnancy in our unit have been published before [2]. Briefly, the diagnosis of EP was made when a well-defined adnexal mass was seen separate from the uterus and the ovary, which had typical morphological characteristics of a tubal EP (Figs 1 and 2). Morphological features of EPs were classified in three groups: gestational sac containing a live embryo, an empty gestational sac with or without a yolk sac and a solid hyperechoic swelling.

Blood was taken for initial serum β hCG measurement from all clinically stable women with ultrasound findings conclusive of a non-viable tubal EP and with no evidence of intra-abdominal bleeding. Non- viable tubal EP was defined as an EP without embryonic heartbeat in the transvaginal ultrasound. Women with viable EP with an embryonic heartbeart were excluded. Serum β hCG were expressed in IU/L according to the World Health Organization, Third International Reference 75/537.

Women who had an initial (day 0) β hCG measurement below 5000 IU/L and remained asymptomatic were offered expectant management. This comprised of serial serum β hCG measurements, on an outpatient basis, until the serum concentration declined to less than 20 IU/L or the urine pregnancy test became negative. All β hCG measurements were recorded in our computerized database, which has been in use since 1998.

Expectant management was discontinued if women developed increasing abdominal pain or the β hCG levels showed sustained rise on repeated measurements. All women with failed expectant management were offered surgery. Surgery was also offered to all women with viable EP and those with an EP who presented with a serum β- hCG more than 5000 IU/l.

Surgical treatment involved laparoscopy in most cases. Medical management was only used selectively in women taking part in ongoing research studies. Women with non-diagnostic scans (pregnancies of unknown location) were not included in this data analysis.

Our database was searched to identify all women diagnosed with tubal EP. The management plan and the treatment outcomes were recorded in all cases. Women who underwent successful expectant management (without need for medical and/or surgical intervention) for a confirmed EP were identified and their serum β hCG measurements recorded (n = 161). In women who had more than two serum β hCG measurements recorded (n = 130), the serum β hCG levels were expressed as percentages of the maximum serum β hCG concentration and plotted against the number of days of follow up, to establish clearance curves.

Patients were further divided into two groups: (group 1) with a steady decline of serum β hCG levels from day 0 onwards (n = 97) and (group 2) with plateaued serum β hCG levels before decline (n = 33).

IRB approval was waived by the Ethics Committee (King’s College Hospital, London, UK).

### Statistical methods

**Fig 3:**
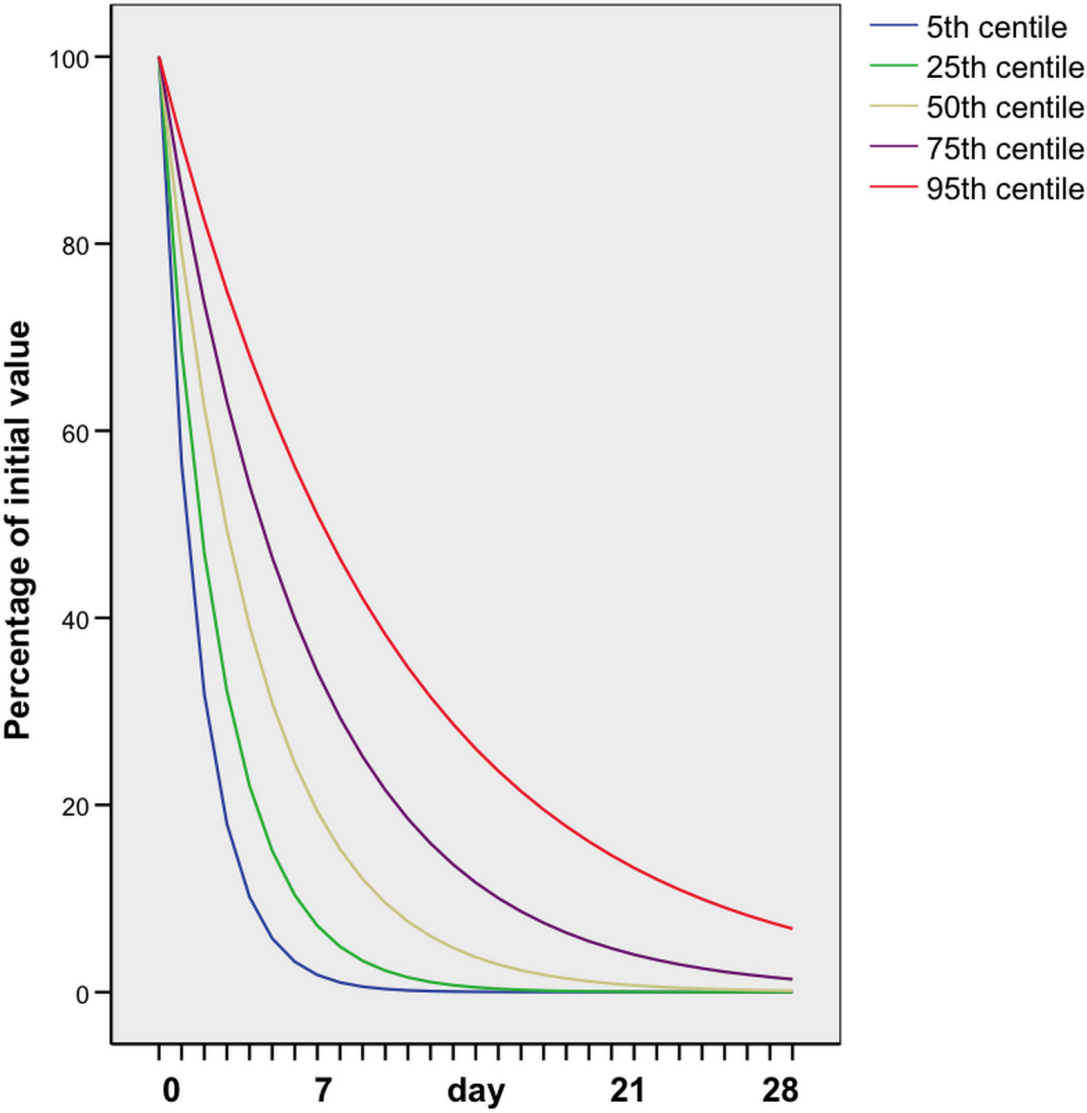
β hCG clearance curves in women with an ectopic pregnancy managed successfully expectantly in whom the β hCG declined steadily from the initial measurement.

**Fig 4:**
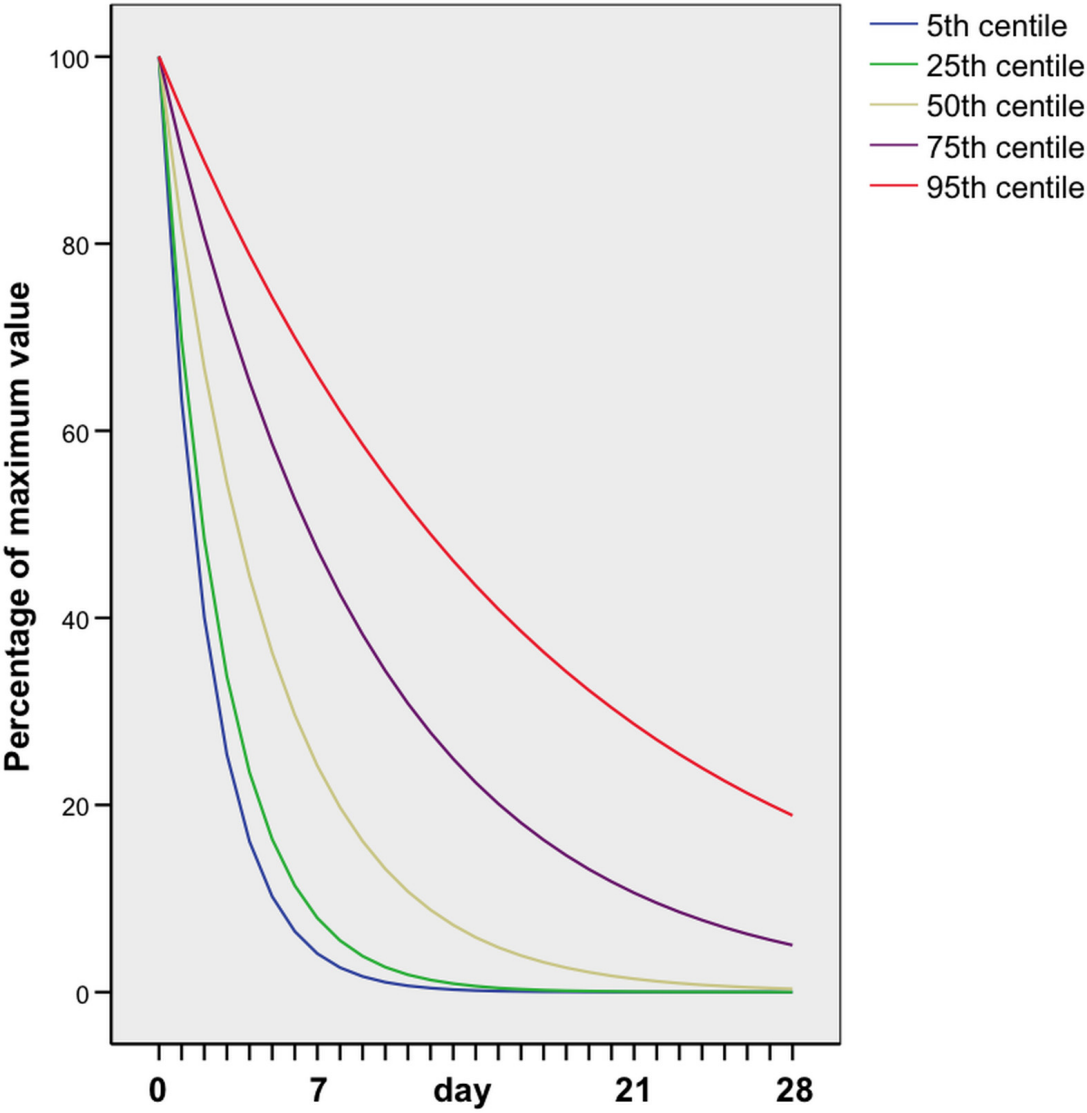
β hCG clearance curves in women with an ectopic pregnancy managed successfully expectantly in whom the β hCG plateaued before declining.

SPSS version 14 was used for the statistical calculations. The two groups were compared with t-test and Chi square test, as appropriate. An exponential curve was fitted to the β hCG clearance for each case that showed sustained decline. For those cases in which β hCG clearance peaked and plateaued before declining, an exponential curve was fitted to the declining phase. For each of the first 28 days from the peak, fifth centiles, ninety-fifth centiles and the quartiles of the fitted values were calculated for group 1 (Fig 3) and group 2 (Fig 4).

## Results

Between December 1998 and July 2006, 696 tubal EPs were diagnosed in our unit. 417 patients (60%) had immediate surgical treatment, 266 patients (38%) underwent expectant management and 13 patients (1.9%) received methotrexate treatment. Of the 266 patients managed expectantly, 161 (61%) were successful, 102 (38%) required surgical intervention whilst 3 (1%) were lost to follow up.

The mean age of women who had successful expectant management was 30 years (range 16 to 42 years). This was not significantly different from the mean age of women treated surgically, both initially and as a result of failed expectant management (mean 30 years, range 14 to 44 years), p>0.05.

In the 161 patients who had successful expectant management the mean initial serum β- hCG was 488 IU/L (range from 41 to 4883 IU/L) and the median serum β hCG clearance time was 19 days (range from 5 to 82 days). Of the 161 patients, 130 (81%) had more than two serum β hCG measurements recorded, 31 (19%) had an initial measurement plus one more.

In the 130 women who had more than two β hCG measurements, two distinct groups were identified in terms of the pattern of β hCG clearance. In group 1, which comprised 97 women (75%), the serum β hCG level showed a sustained decline at a steady rate from the day of initial serum β hCG measurement onwards (Fig 3). In group 2, consisting of 33 women (25%), serum β hCG plateaued for a median of 9 days (range 2 days to 26 days) before starting to decline (Fig 4).

There were no significant differences in women’s age between group 1 and group 2 (29 years [range 16 to 42] vs. 32 years [range 18 to 40]) (p>0.05). The mean diameter of the EPs of group 1 was 18 mm (range 4 to 40) whilst the mean diameter for EPs of group 2 was 17 mm (range 6 to 57) (p>0.05). The morphological characteristics of EPs in the two groups were also similar (Table 1).

**Table 1:** There is no significant difference in the morphology of ectopic pregnancy between the two patterns of β hCG clearance.

	Group 1 (n = 97)	Group 2 (n = 33)	p-value
Maternal age (years) mean;range	30 (16–42)	32 (18–40)	ns
Gestational age at diagnosis (weeks) mean;range	6^+2^ (4^+5^–11^+5^)	6^+3^ (4^+3^–10^+2^)	ns
Ectopic pregnancy diameter (mm) mean; range	18 (4–40)	17 (6–57)	ns
Initial serum beta-hCG (IU/ml) mean;range	463 (143–2184)	569 (41–4883)	ns
Solid tubal swelling (%) 95%CI	79 (71.3–87.4)	76 (61.4–90.6)	ns

Demographic data of patients and morphological characteristics of ectopic pregnancies with successful expectant management. Group 1 (steady decline of serum β hCG levels from day 0 onwards (n = 97)); Group 2 (plateaued serum β hCG levels before decline (n = 33)).

In group 1 the average half life of β hCG clearance was 82.5 hours (±SD 50.2). In group 2 the average half life of β hCG clearance, in the decline phase of the curve, was 106.7 hours (±SD 72.0). However, these differences were not significant (p>0.05).

## Discussion

The median time for β hCG clearance with expectant management of tubal EP in our study (19 days, range 5 to 82 days) was similar to that previously reported for women managed with systemic methotrexate (19 days range 2 to 53) [10], but it was slightly longer than clearance time for women managed by laparoscopic salpingostomy (14 days, range 2 to 50) [10]. Women with plateauing β hCG are often offered treatment with methotrexate in order to facilitate resolution of pregnancy [13]. The result of our study indicates that this may not be necessary as the resolution time of expectant management would not appear to be significantly longer compared to methotrexate. However, women managed with methotrexate may have had higher initial β hCG levels than the women in our study that were managed expectantly. Therefore a formal comparison of methotrexate versus expectant management is necessary to assess potential benefits of methotrexate over expectant management in terms of faster pregnancy resolution.

In our study, EPs successfully expectantly managed showed two distinctive types of behavior. In the majority of cases the β hCG levels steadily declined from the initial visit until the complete resolution of pregnancy. However, in approximately 25% of women with EPs, who underwent successful expectant management, β hCG levels plateaued for up to nearly a month before starting to decline. This difference in behavior of EP was not accompanied by differences in maternal age, size or morphological characteristics of the EP. In view of this it is likely, that differences in the dynamics of βhCG levels are caused by the timing of diagnosis rather than intrinsic differences between EPs, which are destined to resolve spontaneously. In the natural history of tubal miscarriage there is a period of progressive development followed by cessation of growth and ultimately by a phase of regressive changes leading to resolution. It is inevitable that there is significant variability in the length of each phase in individual cases of EPs. It is therefore likely that the majority of cases diagnosed were detected in the phase of resolution and the remaining cases in the phase of stalled development. We do not have cases, which were detected in the early phase of progressive development because our criteria for intervention were partly based on the observation of β hCG changes. Traditionally, intervention is offered to women with EPs and rising β hCG regardless of their clinical symptoms. Our data suggests that the success of expectant management could be increased if intervention in women with non- viable EPs is based on clinical symptoms rather than the monitoring β hCG changes. However, it is impossible at present to differentiate EPs with rising β hCG levels, which resolve spontaneously from those, which are likely to cause clinical symptoms. Therefore further work on improving the selection criteria is necessary before expectant management could be offered to women with EPs and rising β hCG levels. In this study it remains unclear which patient group will fail to have successful expectant management and needs secondary surgery.

Our data also show that plateauing β hCG levels per se should not be used to intervene in asymptomatic women with ectopic pregnancies as a significant number of them will resolve without need for any intervention. However, expectant management with plateauing β hCG levels is longer compared to women with declining β hCG and some women may request intervention simply to reduce the length of follow up.

Once β hCG levels start to decline they tend to clear with a half time of approximately 4 days. The rate of clearance is not different in women with previously plateauing β hCG levels. Declining β hCG levels do not always indicate that expectant management will be successful. However once a declining trend is established, the length of time necessary for spontaneous resolution can be estimated.

### Conclusions

Information about β hCG clearance rates (median follow-up length of 19 days) in tubal EP with expectant management can support women who need to make necessary social arrangements to attend for follow up visits. It may furthermore help doctors to rationalize the number of follow up visits, keeping in mind that women with initially plateauing serum β hCG levels showed a longer follow-up time until β hCG clearance compared to women with steadily declining β hCG levels. However, we are aware of the limitation that our results apply to our specific study population and may not be generalized to all patients.
